# Indirect and Potential Impacts of the COVID-19 Pandemic on the Public Health

**DOI:** 10.34172/jrhs.2020.25

**Published:** 2020-09-30

**Authors:** Abedin Saghafipour

**Affiliations:** ^1^Department of Public Health, Faculty of Health, Qom University of Medical Sciences, Qom, Iran

## Dear Editor-in-Chief

Acute Respiratory Syndrome Coronavirus (SARS-CoV-2), is a zoonotic disease emerged in Dec 2019 and caused the current COVID-19 pandemic^1^. The main mode of transmission of coronavirus is through close person-to-person contact of respiratory droplets from infected people via sneezing and coughing. In addition to respiratory droplets expelled by infected individuals, the corona virus is transmitted through contaminated objects and through the air, especially in closed and poorly ventilated environments^2,3^.

The World Health Organization recommends measures such as social distancing, regular hand washing, use of masks and gloves, greater sensitivity in disinfecting fruits and vegetables, disinfection of surfaces, and full personal hygiene to prevent human infection with Covid-19^4,5^.

The potential impacts of the Covid-19 pandemic on the public health of individuals in the community can be imagined. Some common examples adverse effects related to Covid-19 pandemic are as follows: It seems, due to the fear of the possibility of transmitting coronavirus in health care centers, people likely come less to taking health care, diagnosis and treatment of non-communicable diseases such as cardiovascular disease in hospitals and clinics. Furthermore, routine vaccination coverage of children aged <6 yr might be reduced.

On the other hand, there is a possibility of misdiagnosis of Covid-19 instead of many other infectious diseases whose initial symptoms are similar to it. Besides, due to the deaths caused by this disease and the restrictions on the movement of people and the decrease in visits of families and acquaintances, the mental health status of people in the community may be shaken.

As a result of improper use of some disinfectants used to disinfect surfaces and foodstuffs, there is a possibility of potentially increasing poisoning. In addition, excessive use of alcohol and detergents will cause skin disorders. Finally, due to traffic restrictions and home quarantine at home, there is a possibility of weight gain and obesity in people, which will lead to many non-communicable diseases. It seems that the Covid-19 pandemic and the application of preventive measures in some cases have prevented other diseases and reduced their health consequences. For instance, using a mask to prevent Covid-19 can also reduce the risk of other respiratory diseases, such as the flu and tuberculosis^6^. The mask can also reduce the adverse effects of air pollution^7^. In addition, social distancing may reduce the incidence of skin diseases such as head lice infestation in individuals, especially among students. Hand washing is a way to prevent food and waterborne diseases such as cholera and can reduce the transmission of these diseases^8^.

## Conflict of interest

The authors declare that there is no conflict of interest.

## References

[1] Rodriguez-Morales AJ, Cardona-Ospina JA, Gutiérrez-Ocampo E, Villamizar-Peña R, Holguin- Rivera Y, Escalera-Antezana JP (2020). Clinical, laboratory and imaging features of COVID-19: A systematic review and meta-analysis. Travel Med Infect Dis.

[2] Chan JFW, Yuan S, Kok KH, Kai-Wang K, Chu H, Yang J (2020). A familial cluster of pneumonia associated with the 2019 novel coronavirus indicating person-to- person transmission: A study of a family cluster. Lancet.

[3] Cao Z, Li T, Liang L, Wang H, Wei F, Meng S (2020). Clinical characteristics of Coronavirus Disease 2019 patients in Beijing, China. PLoS One.

[4] Mohler G, Bertozzi AL, Carter J, Short MB, Sledge D, Tita GE (2020). Impact of social distancing during COVID-19 pandemic on crime in LosAngeles and Indianapolis. J Crim Justice.

[5] Ma QX, Shan H, Zhang HL, Li GM, Yang RM, Chen JM (2020). Potential utilities of mask-wearing and instant hand hygiene for fighting SARS-CoV-2. J Med Virol.

[6] Chughtai AA, Khan W (2019). Use of personal protective equipment to protect against respiratory infections in Pakistan: A systematic review. J Infect Public Health.

[7] Carlsten C, Salvi S, Wong Gwent al (2020). Personal strategies to minimise effects of airpollution on respiratory health: advice for providers, patients and the public. Eur Respir J.

[8] Taylor DL, Kahawita TM, Cairncross S, Ensink JH (2015). The Impact of Water, Sanitation and hygiene interventions to control cholera: a systematic review. PLoS One.

